# Outcome of Surgical Intervention for Intrathoracic Lymph Node Metastasis in Uterine and Ovarian Cancer without Lung Metastasis: A Report of Three Cases

**DOI:** 10.70352/scrj.cr.24-0080

**Published:** 2025-01-31

**Authors:** Ryohei Miyazaki, Masaya Tamura, Marino Yamamoto, Hironobu Okada, Yusuke Ujihara, Takashi Ushiwaka, Nagamasa Maeda

**Affiliations:** 1 Department of Thoracic Surgery, Kochi Medical School, Kochi University, Nankoku, Kochi, Japan; 2 Department of Obstetrics and Gynecology, Kochi Medical School, Kochi University, Nankoku, Kochi, Japan

**Keywords:** lymph node metastasis, surgical intervention, thorax, uterine and ovarian cancer

## Abstract

**INTRODUCTION:**

Metastasis to the hilar and mediastinal lymph nodes in gynecological cancer is rare, and isolated hilar or mediastinal lymph node metastases are even rarer. In this report, we describe the results of lymph node dissection performed on 3 patients with hilar mediastinal lymph node metastasis but no lung metastasis from uterine or ovarian cancer.

**CASE PRESENTATION:**

Case 1 was a 50-year-old woman diagnosed with ovarian cancer with mediastinal lymph node metastasis. After 4 courses of chemotherapy, a total hysterectomy, omentectomy, and mediastinal lymph node dissection were performed simultaneously. The patient is still alive 58 months after surgery. Case 2 was a 68-year-old woman who underwent a total hysterectomy after chemotherapy for endometrial cancer with multiple lymph node metastases. Forty-two months after surgery, mediastinal lymph node dissection was performed for metastasis of uterine cancer. She is still alive 75 months after surgery. Case 3 was a 69-year-old woman who underwent a hysterectomy for endometrial cancer. One year after surgery, she underwent thoracoscopic hilar and mediastinal lymph node dissection due to metastasis. Thirty-nine months have passed with no recurrence. Aggressive local control, particularly surgical resection of isolated hilar mediastinal lymph nodes in gynecological cancer, may contribute to prolonging patient survival.

**CONCLUSIONS:**

Aggressive local control, especially surgical resection, for isolated hilar mediastinal lymph nodes due to gynecological cancer is safe and may contribute to prolonging survival.

## Abbreviations

PET
: positron emission tomography
FDG
: fluorodeoxyglucose
SBRT
: stereotactic body radiation therapy
DFS
: disease-free survival
OS
: overall survival

## INTRODUCTION

There are few cases in which hilar or mediastinal lymph node metastasis is found during the treatment of malignant tumors outside the chest. Most of these cases involve lung metastasis,^1–3)^ while cases with only hilar and mediastinal lymph node metastases in the thoracic cavity are even rarer. Several studies have reported the use of chemoradiotherapy for hilar and mediastinal lymph node metastatic lesions in gynecological cancer, but there is insufficient data on surgical resection and its therapeutic effects in these cases.

In this report, we describe the results of lymph node dissection procedures that were performed in 3 patients with uterine or ovarian cancer and hilar mediastinal lymph node metastasis but no lung metastasis.

## CASE PRESENTATION

### Case 1

**Fig. 1:**
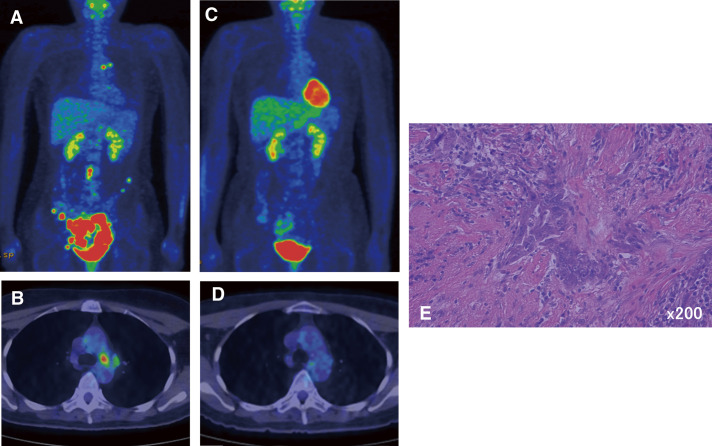
PET scans before (**A, B**) and after chemotherapy of Case 1 (**C, D**) demonstrated the disappearance of the left mediastinal metastasis. However, hematoxylin and eosin staining (×200) of the mediastinal lymph node revealed the presence of residual malignant cells (**E**). PET, positron emission tomography

A 50-year-old woman was diagnosed with ovarian cancer, intraperitoneal dissemination, para-aortic lymph node metastasis, and left mediastinal lymph node metastasis (**Figs. 1A** and **1B**) after bilateral salpingo-oophorectomy during an exploratory laparotomy. After 4 courses of chemotherapy using paclitaxel, carboplatin, and avastin, a positron emission tomography (PET) scan showed that fluorodeoxyglucose (FDG) uptake in the tumor decreased remarkably (**Figs. 1C** and **1D**). We diagnosed that chemotherapy was effective and performed salvage surgery. A total hysterectomy, omentectomy, pelvic, and para-aortic lymph node dissection, and left mediastinal lymph node dissection were performed simultaneously. We performed mediastinal lymph node dissection of #4L, #5, and #6. In histological findings, no residual tumor was found in the uterus or omentum, but metastatic cells were found in the right para-aortic lymph nodes and mediastinal lymph nodes (**Fig. 1E**). After surgery, 6 additional courses of chemotherapy were administered and the patient was followed up. The patient developed a recurrence of peritoneal dissemination 35 months after surgery. Therefore, we changed the regimen and resumed chemotherapy, and she is still alive 58 months later.

### Case 2

**Fig. 2:**
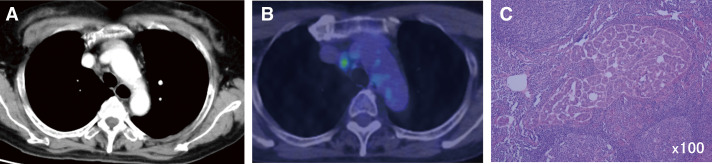
Contrast-enhanced CT scan (**A**) and PET scan (**B**) of Case 2 show right mediastinal lymphadenopathy. (**C**) Hematoxylin and eosin staining (×100) of the mediastinal lymph node revealed the presence of malignant cells. CT, computed tomography; PET, positron emission tomography

A 68-year-old woman underwent chemotherapy for endometrial cancer with multiple lymph node metastases in the groin and periaortic, axillary, and cervical regions. Due to significant tumor shrinkage, she underwent a total hysterectomy, bilateral salpingo-oophorectomy, and pelvic lymph node dissection. Histological findings showed the complete response of the para-aortic lymph nodes. Forty-two months after surgery, we found a mediastinal lymph node with a tendency to grow, measuring 1.0 cm, and the lymph node had FDG uptake with a maximum standard unit value of 5.1 (**Figs. 2A** and **2B**). A thoracoscopic lymph node biopsy revealed metastasis of uterine cancer (**Fig. 2C**); thus, we performed mediastinal lymph node dissection of #4R and #2R. After surgery, 3 additional courses of chemotherapy were administered, and the patient was followed up. After 33 months of lymph node dissection, bilateral mediastinal lymph node enlargement was also observed. She underwent a thoracoscopic subcarinal lymph node biopsy and was diagnosed with a sarcoid-like reaction. After 63 months of the initial dissection, the patient was diagnosed with cervical lymph node metastasis. We resumed chemotherapy after resection of the cervical lymph nodes. She has been alive for 75 months after surgery.

### Case 3

**Fig. 3:**
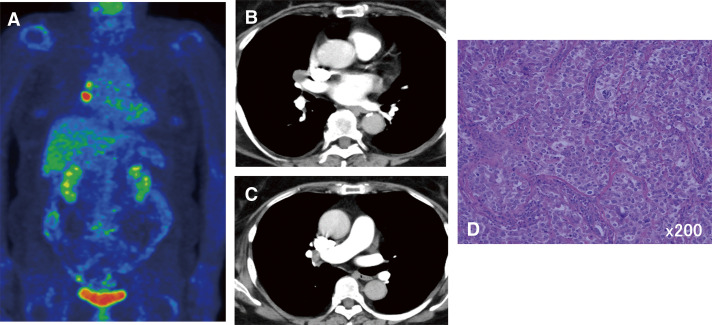
PET scan (**A**) and contrast-enhanced CT (**B, C**) scans of Case 3 show right hilar lymphadenopathy. Hematoxylin and eosin staining (×200) of the hilar lymph node revealed the presence of malignant cells (**D**). CT, computed tomography; PET, positron emission tomography

A 69-year-old woman underwent a subradical hysterectomy, bilateral salpingo-oophorectomy, pelvic lymph node dissection, and omentectomy for stage II endometrial cancer. Histological examination revealed no lymph node metastasis in the pelvis or abdominal cavity. One year after surgery, she developed right hilar lymph node enlargement, and she was referred to our department. PET scans revealed enlarged lymph nodes with FDG uptake in #12m and #11s (**Figs. 3A–3C**). She underwent a thoracoscopic biopsy of #12m and was diagnosed with metastasis (**Fig. 3D**); thus, we performed additional hilar and mediastinal lymph node dissection of #12m, #11s, #10, #4R, and #2R. After surgery, 3 additional courses of chemotherapy were administered, and the patient was followed up. Six months after surgery, she was diagnosed with brain metastasis and underwent radiotherapy. After radiation therapy, she was followed up without chemotherapy. Thirty-nine months have passed since her lymph node dissection, and she has had no recurrence, even in the hilar mediastinal lymph nodes.

## DISCUSSION

Metastasis to the hilar and mediastinal lymph nodes in gynecological cancer is rare, and isolated hilar or mediastinal lymph node metastases are even rarer. The underlying mechanisms and routes for solitary hilar and mediastinal lymph node metastasis in gynecological cancer are not completely understood but may include the following: (i) An extension from the diaphragmatic crus and para-aortic nodes, followed by mediastinal and intrapulmonary lymph node metastasis, with an antegrade route that connects to the thoracic duct and (ii) A route that connects liver metastases to the thoracic lymphatic vessels.^2–5)^ In our study, para-aortic lymph node metastasis was observed in both cases 1 and 2, suggesting the existence of the first route mentioned above. In the third case, no lymph node metastasis was observed, and although the route is unknown, the brain metastasis developed early, indicating the possible involvement of hematogenous metastasis.

There are a small number of reports regarding the treatment of isolated hilar and mediastinal lymph node metastases. In 2 cases, solitary mediastinal lymph node metastasis was found at the time of diagnosis of cervical cancer and treated only using palliative therapy with prognoses of 9 months and 11 months, respectively.^6)^ There are multiple reports of radiation-based treatment for isolated hilar or mediastinal lymph node metastases in gynecological cancer. In the KROG 14-11 trial, 1 out of 85 patients had mediastinal oligometastatic disease. With stereotactic body radiation therapy (SBRT), this patient achieved her disease-free survival (DFS) of 26.7 months.^7)^ Bonilla et al.^8)^ treated 2 patients with isolated mediastinal lymph node recurrence after cervical cancer surgery and reported DFS of 33 and 49 months, respectively. In addition, Ning et al.^9)^ published a report on chemoradiation treatment of 38 patients with oligometastatic recurrence, including 10 cases of isolated mediastinal metastases for cervical cancer. They reported a median DFS of 21.7 months, a median overall survival (OS) of 50.7 months, and a 2-year OS of 73.5%.

Although the safety of surgical treatment for intrathoracic tumors, including mediastinal lymph node resection, in ovarian cancer has been established, its effectiveness remains unclear.^10)^ Kawaguchi et al.^11)^ performed left hilar and mediastinal dissection for intrathoracic lymph node metastasis 26 months after radical treatment for cervical cancer, and then conducted right hilar lymph node dissection 25 months later. She developed retroperitoneal recurrence and died 8 months after the second lymph node dissection.

In one study of 11 cases with extrathoracic cancer that did not include gynecological cancer, metastasis was found only in the hilar and mediastinal lymph nodes; thus, these patients underwent resection, and good results were obtained with a 5-year survival rate of 41.6%.^12)^ In our study, cases 1 and 2 have not shown any recurrence of lesions for more than 2 years after lymph node dissection, while the third patient (Case 3) has brain metastasis but is currently alive and has not exhibited recurrence. In addition, local control was achieved in all 3 cases.

If lymph node metastasis around the trachea or bronchus progresses, it may invade the bronchi or trachea,^13,14)^ leading to airway bleeding and stenosis, so local control is important. While surgery after radiotherapy can be difficult, radiotherapy after surgery is relatively feasible. If the patient’s general condition permits, surgical therapy for isolated resectable hilar or mediastinal lymph node metastases may be meaningful in the context of avoiding radiotherapy.

## CONCLUSION

In conclusion, aggressive local control, especially surgical resection, for isolated hilar mediastinal lymph nodes in gynecological cancer is safe and may contribute to prolonging patient survival.

## ACKNOWLEDGMENTS

Not applicable.

## DECLARATIONS

### Funding

None declared.

### Authors’ contributions

Ryohei Miyazaki wrote and edited the manuscript.

Masaya Tamura proposed an idea and reviewed the manuscript.

All authors performed surgical procedures and/or participated in patient care.

All authors read and approved the final manuscript and agree to be responsible for all aspects of the study.

### Ethics approval and consent to participate

The authors declare that appropriate written informed consent was obtained from the patients for the publication of this case report and any accompanying images. Informed consent was waived because of the retrospective nature of the study.

### Consent for publication

We obtained consent for publication from the patient.

### Competing interests

The authors declare that there are no competing interests regarding the publication of this article.
